# Enhancing meniscal repair with tough adhesive puncture sealing (TAPS) suture: A proof‐of‐concept study on bovine cadaveric knees

**DOI:** 10.1002/jeo2.70129

**Published:** 2024-12-30

**Authors:** Stephanie Lamer, David Mazy, Marie‐Lyne Nault

**Affiliations:** ^1^ Department of Surgery University of Montreal Montréal Québec Canada; ^2^ Azrieli Research Center CHU Sainte‐Justine Montréal Québec Canada; ^3^ Department of Orthopedic Surgery CIUSSS Nord de l'Ile Hôpital du Sacré‐Coeur de Montréal (HSCM) Montreal Québec Canada

**Keywords:** adhesive hydrogel, cadaveric study, coated suture, meniscal repair, meniscal suture

## Abstract

**Purpose:**

The objective was to use cyclic tensile loading to compare the gap formation at suture site of three different suture materials to repair bovine radial meniscal tears: (1) polyglactin sutures, (2) tough adhesive puncture sealing (TAPS) sutures and (3) ultra‐high molecular weight polyethylene (UHMWPE) sutures.

**Methods:**

Twelve ex vivo bovine knees were dissected to retrieve the menisci. Complete radial tears were performed on 24 menisci, which were then separated into three groups and repaired using either pristine 2–0 polyglactin sutures, TAPS sutures (2–0 polyglactin sutures coated with adhesive chitosan/alginate hydrogels) or 2‐0 UHMWPE sutures with a single stitch. The repaired menisci were clamped onto an Instron machine and underwent cyclic loading between 5 and 25 N at a frequency of 0.16 Hz. Gap formation between the edges of the tear was measured after 500 cycles using an electronic caliper, when the meniscus was still on the Instron without any load applied.

**Results:**

Mean gap formation was 5.22 mm (±1.70) for the 2–0 polyglactin sutures, 2.48 mm (±0.25) for the TAPS sutures, and 4.85 mm (±1.55) for the 2–0 UHMWPE sutures. The gap was significantly smaller in the TAPS sutures group compared to the two others because of better force dispersion, decreasing tissue damage by suture indentation and potentially leading to better meniscal healing.

**Conclusions:**

From a biomechanical standpoint, coated sutures held the edges of radial meniscal tears closer together compared to conventional sutures. This technology has the potential to reduce tissue damage and improve the success rate of meniscal repairs.

**Level of Evidence:**

controlled laboratory study.

**Abbreviations**
EDC: 1‐ethyl‐3‐(‐3‐dimethylaminopropyl) carbodiimide hydrochloride
NHS: N‐hydroxysuccinimide
TAPS: tough adhesive puncture sealing
UHMWPE: ultra‐high molecular weight polyethylene

## INTRODUCTION

The meniscus is an indispensable and crucial component of the knee joint, playing a role in load‐bearing, shock absorption, stabilization, proprioception and lubrication [6, 20, 27, 35, 41]. Meniscal tears are the most common knee injury, occurring either acutely or as a result of degenerative conditions [6, 27, 41]. Meniscectomy or meniscal repair are the two surgical options available to treat meniscal tears [27, 41]. However, meniscal suture repair is a challenging and time‐consuming surgery [13, 38] that requires a longer rehabilitation period compared to meniscectomy [12]. Conversely, meniscectomy is easier, faster and allows patients to return to their activities sooner [9, 39]. Nevertheless, it has long‐term consequences and can cause degenerative changes in the knee, leading to early‐onset osteoarthritis and the potential need for a knee replacement [8, 16, 18, 27, 35, 41]. For these reasons, meniscal repair is often preferred in young patients, especially considering that even partial meniscectomy can cause radiological changes compatible with osteoarthritis [10, 28].

Among the many devices developed to hold together the edges of a meniscal tear (sutures, stingers, arrows and darts), the current gold standard is suture repair [4, 6]. However, meniscal repair is challenging, since the knee joint provides little space to work in and tear morphology changes from one person to the other [3].

Failure rates vary widely from one study to the next, but the reported failure rate is approximately 25% [1, 30, 42, 33]. Reasons for failure include tissue damage and stresses being concentrated in specific areas where sutures pass through the meniscus. Additionally, the rough, braided suture surface can cause shearing forces when in contact with dynamic tissue, potentially leading to meniscal repair failure [4, 29].

Thanks to rapidly evolving advances in bioengineering, new tissue adhesives to repair meniscal tears were developed and tested over the years [6, 7]. Unfortunately, a systematic review by Marom et al. in 2021 concluded that none yet were ideal for meniscal repair [25]. Nonetheless, these biomaterials still have the potential to improve outcomes and tissue healing when biological compounds such as cells, growth factors, platelet‐rich‐plasma and enzymes are added to its composition, playing an adhesive structural role but also acting like a scaffold to carry those molecules [6, 18]. Therefore, the first step to develop those complete biomaterials, [32] is to find a composition that is adequate for the meniscus' environment and that is at least as strong as the material now used to repair menisci. In terms of functional, socioeconomic and clinical impact, the idea of developing an adhesive that could facilitate and encourage meniscal repair is very attractive. Hence, a new resistant adhesive hydrogel was developed and tested in bovine menisci [22, 24].

The main hypothesis was that tough adhesive puncture sealing (TAPS) sutures have greater tensile strength than standard sutures and ultra‐high molecular weight polyethylene (UHMWPE) sutures, resulting in smaller gaps between the edges of the tear. The secondary hypothesis was that gap formation at the tear site would be the result of the indentation of the uncoated sutures into the meniscal tissue.

The main objective of this study was to compare the biomechanical properties of three sutures for the repair of radial meniscus tears on bovine menisci specimens: (1) standard polyglactin suture, (2) TAPS suture and (3) UHMWPE suture with cyclic tensile tests. The secondary objective was to identify the cause of gap formation.

## MATERIALS AND METHODS

A controlled laboratory study was conducted using 12 fresh bovine knees from the mid femur to mid tibia. The menisci were completely dissected and removed from the proximal tibia (24 specimens, 12 medial and 12 lateral menisci). The dissection was performed meticulously, and the specimens were inspected to ensure there was no prior tear. After dissecting the 24 menisci, they were frozen at −70°C for future testing, and then thawed for 24 h before manipulation. Because the bovine knees came from the food industry, there was no animal sacrifice, and, therefore, no need for research ethics committee approval.

Three groups of eight menisci were created to test tensile strength: meniscal tears sutured with (1) 2–0 polyglactin (Vicryl®) sutures, (2) TAPS sutures (2–0 Vicryl® coated with adhesive hydrogels) and (3) 2–0 UHMWPE (HiFi®) sutures.

**Figure 1:**
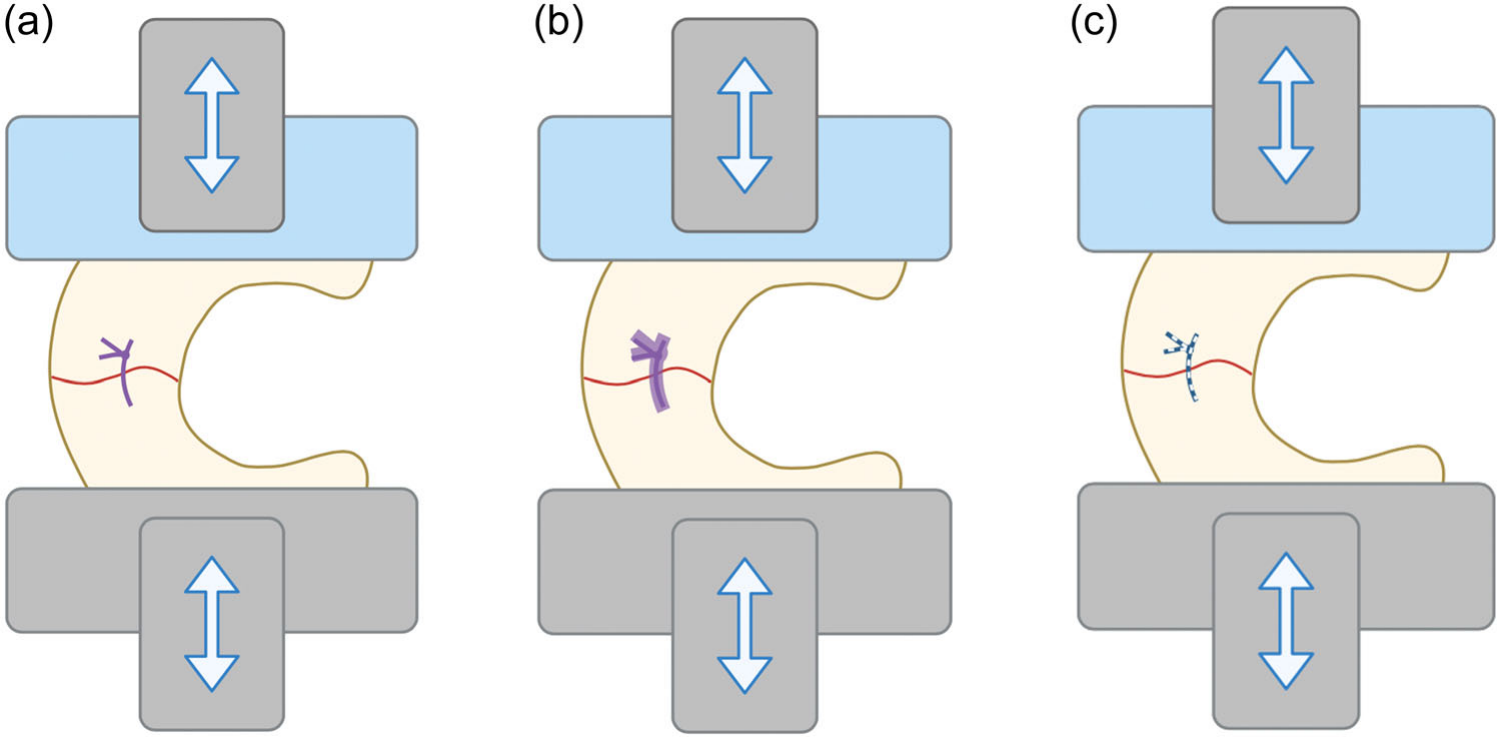
(a) Uncoated polyglactin, (b) tough adhesive puncture sealing, (c) ultra‐high molecular weight polyethylene sutures for complete radial meniscus tear under cycling tensile forces.

Once thawed, a complete radial tear in the middle of each meniscus was done with a scalpel. The radial tear was then repaired using the appropriate suture according to group assignment as defined above, using a single stitch in the middle of the meniscus width and secured with five knots to reduce technical bias (Figure 1).

The TAPS sutures were produced using the process described in the studies published by Ma et al. [23, 24]. The original suture (violet 2–0 polyglactin) was first treated with 1 M NaOH solution to create a carboxylic acid group on the surface. It was then thoroughly rinsed and air dried. At this time, the sutures were considered activated and inserted into a glass capillary tube (World Precision Instrument, TW120‐6). The diameter of the glass tube defined the thickness of the gel sheath. The adhesive solution was composed of coupling reagents, EDC ([1‐ethyl‐3‐(‐3‐dimethylaminopropyl) carbodiimide hydrochloride), and NHS (N‐hydroxysuccinimide), combined with a chitosan solution (chitosan powder [>95%; Lyphar Biotech]). The chitosan solution was injected in the capillary tube with the suture. The prepolymer gel solution composed of acrylamide, alginate, ammonium persulfate and methylene bisacrylamide was then added. Approximately 12 h later, the coated sutures were gently removed from the capillary tubes and placed in a CaCl_2_ solution for storage until use. The preparation time was approximately 2 h. The final dehydration and rehydration steps, which consist of placing the suture over a hot plate and mixing it with a chitosan, EDC and NHS mixture, can be done just before using the suture. The time between these steps and irreversible binding of the suture to the tissue is long enough for it to be easy to manipulate.

**Figure 2:**
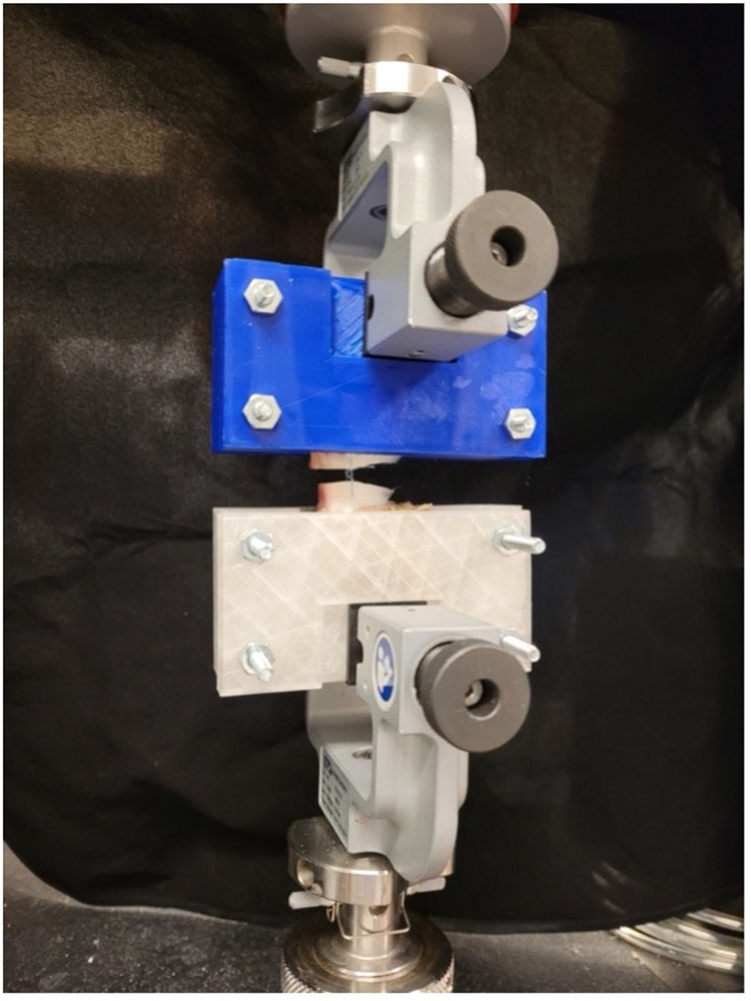
Set‐up of a bovine meniscus with a complete radial tear repaired with pristine suture during tensile testing on the Instron machine.

Each specimen was placed on a universal testing Instron 5965 machine (Instron Inc.). For this protocol, the meniscus needed to be completely removed from the tibia. Both ends of the repaired meniscus were maintained on the testing machine using solid clamps (Figure 2).

**Figure 3:**
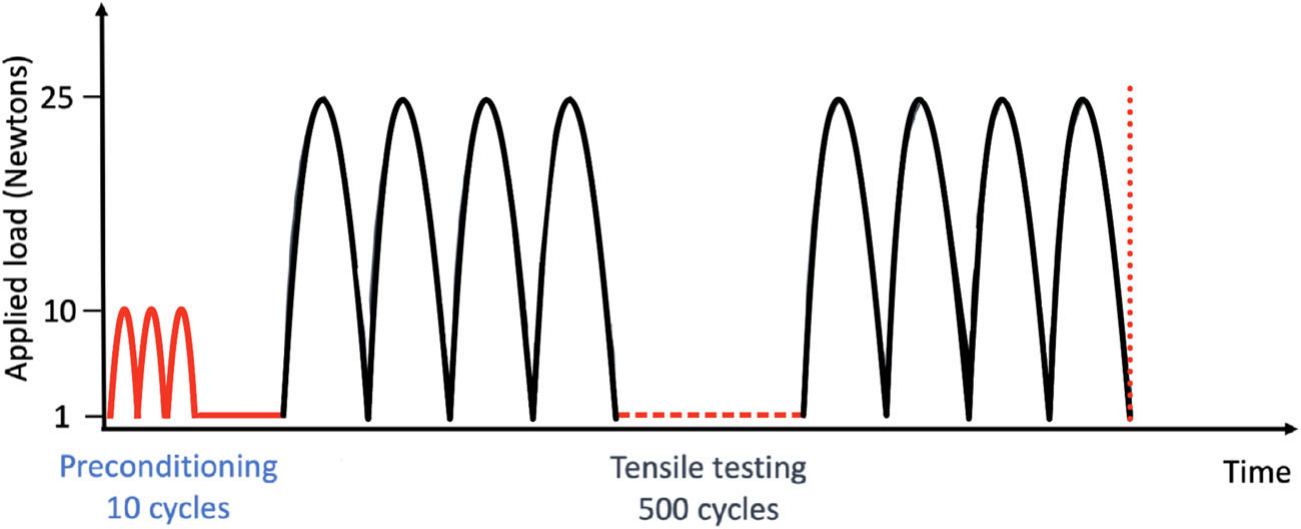
Cyclic loading profile. Preconditioning period of 10 cycles between 1 and 10 N at 0.5 Hz followed by 500 cycles of tensile loading between 5 and 25 N at a frequency of 0.16 Hz.

Tensile testing was then conducted on the repairs. Specimens underwent a preconditioning period of 10 cycles between 1 and 10 N at 0.5 Hz. Then, specimens underwent cyclic tensile loading for 500 cycles between 5 and 25 N at a frequency of 0.16 Hz (Figure 3). Some data suggest that when a human cadaveric knee is loaded with 300 N, the mean distraction force on the meniscus does not exceed 4.7 N [5, 37]. Considering that bovine menisci are thicker and larger than human menisci, we used a range of distraction force going from 5 to 25 N.

Gap formation between the edges of the tear after 500 cycles of tensile testing was measured using an electronic caliper when the menisci were still on the Instron machine without any load. The length of the suture loop was also measured before and after testing to verify that there was no elongation or unknotting of the suture. Before testing, the length was measured once the knot was tied on the meniscus, adding the distance between the two puncture holes on the superior and inferior surface, along with twice the thickness of the meniscus. After the test, it was easier to measure the length of the suture, as the suture was cut cleanly, and the total length of the suture was measured.

For statistical analysis, the three groups were compared using Kruskal–Wallis analysis of variance tests. After confirming that the data was normally distributed, a paired Student's *t* test was conducted to compare the lengths of the suture loops before and after testing. Statistical significance was set to *p* < 0.05. SPSS Statistics (v27.0.1.0) software was used for those analysis.

## RESULTS

**Figure 4:**
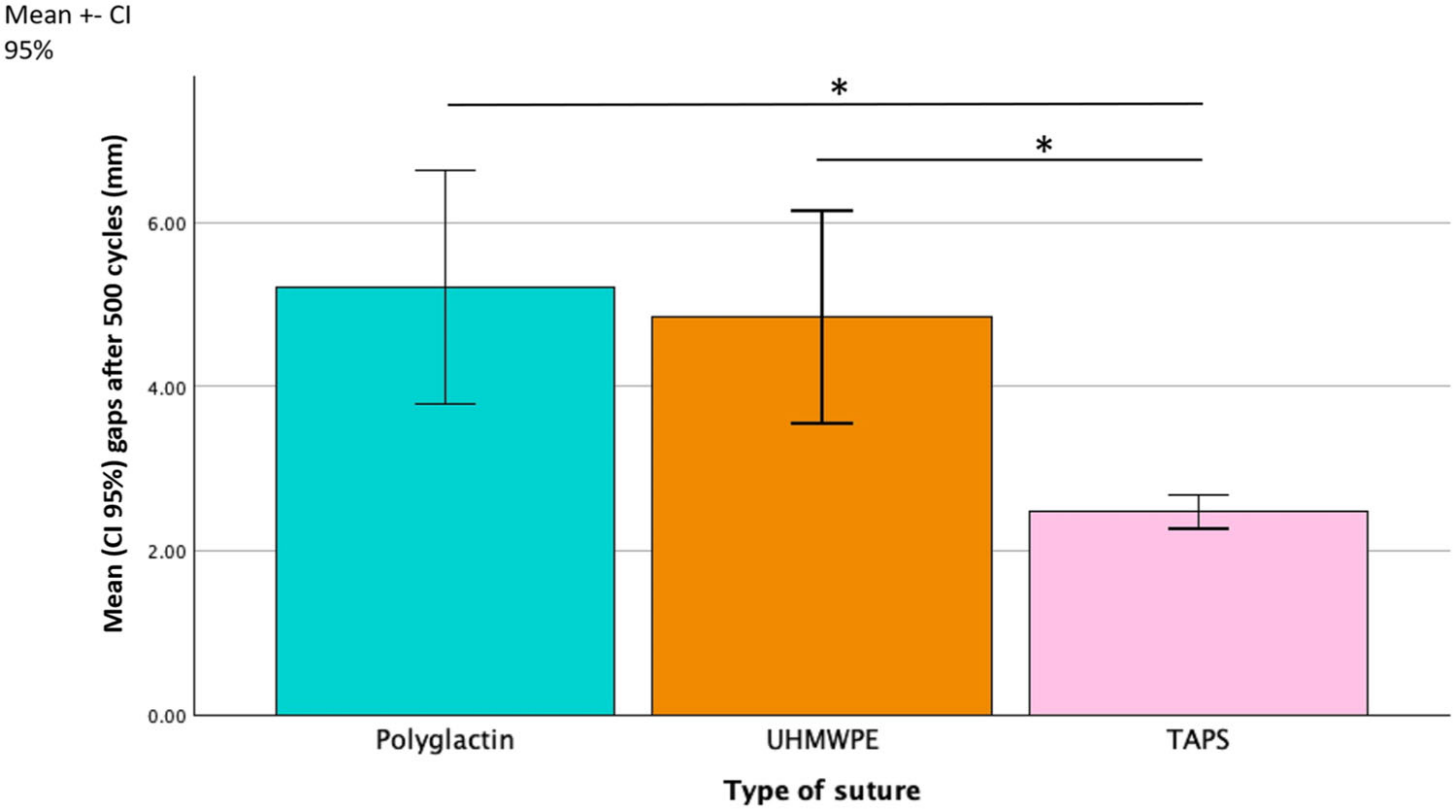
Mean gap formation after 500 cycles of tensile loading. * correspond to *p* < 0.05.

When the bovine meniscus radial tear was repaired with a 2–0 polyglactin suture, the gaps after 500 cycles of tensile testing ranged from 4.03 to 9.20 mm. In the same conditions using the TAPS suture, the gaps ranged from 2.20 to 2.87 mm, and with UHMWPE sutures, the gaps ranged from 2.48 to 6.86 mm. The mean gap formation for polyglactin sutures was 5.22 mm (±1.70) after 500 cycles of tensile testing, 2.48 mm (±0.25) for TAPS sutures, and 4.85 mm (±1.55) for UHMWPE sutures. Comparing the three groups, gap formation with the TAPS sutures was significantly smaller than with standard sutures (UHMWPE vs. TAPS—*p* = 0.002 and polyglactin vs‐. TAPS—*p *= 0.001) (Table 1; Figure 4).

**Table 1:** Gap formation after 500 cycles of tensile loading.

Results after 500 cycles of tensile loading					
Polyglactin—no coating (mm)		TAPS (mm)		UHMWPE—no coating (mm)	
Specimen 1	4.83	Specimen 9	2.33	Specimen 17	6.26
Specimen 2	5.65	Specimen 10	2.67	Specimen 18	4.06
Specimen 3	9.20	Specimen 11	2.38	Specimen 19	2.48
Specimen 4	4.21	Specimen 12	2.20	Specimen 20	3.38
Specimen 5	4.15	Specimen 13	2.31	Specimen 21	5.12
Specimen 6	4.03	Specimen 14	2.32	Specimen 22	6.29
Specimen 7	5.12	Specimen 15	2.87	Specimen 23	4.35
Specimen 8	4.53	Specimen 16	2.74	Specimen 24	6.86
**Mean (±SD)**	**5.22 (**±**1.70)**		**2.48 (**±**0.25)**		**4.85 (**±**1.55)**
**Mean [CI 95%]**	**5.22 [3.79–6.64]**		**2.48 [2.27–2.68]**		**4.85 [3.55–6.15]**
***p* Values**					
Polyglactin vs. TAPS		**0.001**			
TAPS vs. UHMWPE		**0.002**			
UHMWPE vs. polyglactin		0.832			

*Note*: Bold values are statistically significant.

Abbreviations: CI, confidence interval; mm, millimetres; SD, standard deviation; TAPS, tough adhesive puncture sealing; UHMWPE, ultra‐high molecular weight polyethylene.

The length of the suture loop remained consistent before and after tensile testing for the three different suture types. No statistical difference was found when comparing loop length before and after testing (Table 2).

**Table 2:** Loop length before and after 500 cycles of tensile loading.

	Loop length (mm) before testing (mean +‐SD)	Loop length (mm) after testing (mean +‐SD)	*p* Values
Polyglactin	33.5 + −4.2	34 + −3.2	0.582
TAPS	32.5 + −3.3	33.1 + −2.3	0.544
UHMWPE	31.4 + − 4.3	32.8 + −3.8	0.188

Abbreviations: mm, millimetres; SD, standard deviation; TAPS, tough adhesive puncture sealing; UHMWPE, ultra‐high molecular weight polyethylene.

With regard to our second objective, we analyzed the meniscal tissue after tensile testing to determine the failure mechanism. Tissue indentation was always involved in the failure mechanism.

## DISCUSSION

The most important finding of this study is that TAPS suture allows for better force dispersion at the suture site, leading to less tissue damage and a smaller gap between the edges of the tear. Hence, it is possible to hypothesize that this would lead to better meniscal healing in vivo, although further research is needed to confirm this statement. Furthermore, the use of TAPS needs to be evaluated with different sutures and meniscal tear configurations.

Prior studies have shown that although some tissue adhesives are already used in clinical practice [28, 29, 32], none of these options are optimal for meniscal suturing. Essentially, their mechanical properties lack the required adhesive strength and/or show poor biocompatibility [6]. As of now, there are no adhesive components for meniscal repairs available for regular use in a clinical setting, despite past attempts [2, 9, 11, 14, 15, 17, 19, 21, 31, 34, 36, 40].

*This underscores the importance of continued research in this field and the potential uses of our findings*: TAPS sutures outperformed standard sutures during tensile testing. The results demonstrate that gap formation after 500 cycles of tensile testing was smaller when using TAPS sutures compared to polyglactin and UHMWPE sutures. One can hypothesize that this would improve tear healing in an in vivo model, by allowing both ends of the tear to remain in closer proximity. The initial protocol for this study only compared the TAPS and polyglactin sutures. However, after finding a significant difference between these two, we decided to add UHMWPE to the protocol to ascertain whether TAPS sutures would also be stronger than the most commonly used sutures for meniscal repair, which they were.

The length of the suture was equal before and after cyclic tensile testing, meaning that gap formation was not caused by suture elongation or knot loosening (Table 2). Postexperimentation observation revealed that the gap was the result of suture filament indentation in the meniscal tissue. The fact that the gap was smaller with the TAPS sutures indicated a better dispersion of force on the meniscal tissue, meaning the TAPS sutures caused fewer shearing forces on the tissue [23]. Although UHMWPE sutures are known to be very strong, this may not be beneficial for meniscal repair. In fact, there is a biomechanical mismatch between UHMWPE sutures and meniscal tissue, which causes the sutures to cut into the meniscus. This probably contributed to their poorer results compared to TAPS sutures in tensile testing.

**Figure 5:**
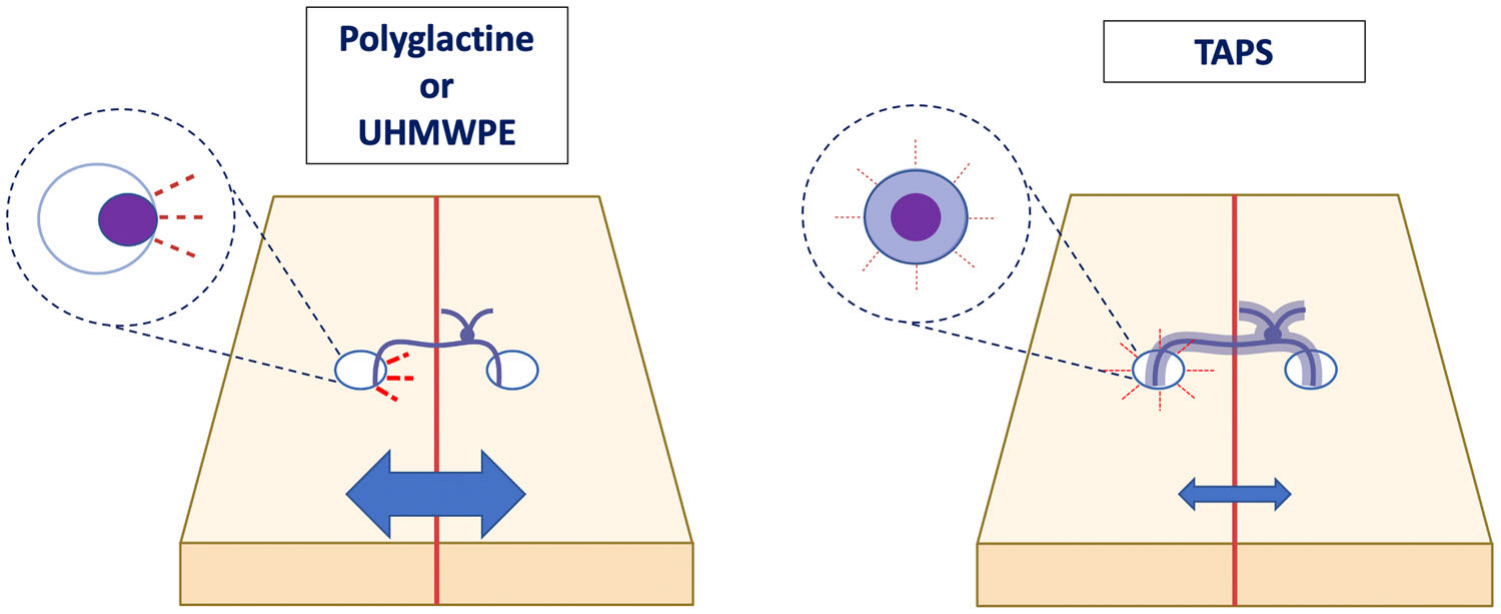
Mechanism by which the coated suture allows better dissipation of applied forces by decreasing biomechanical mismatch, stress concentration, friction and reducing the space formed at the lesion site. TAPS, tough adhesive puncture sealing; UHMWPE, ultra‐high molecular weight polyethylene.

The swelling property of the TAPS sutures is another advantage that could prevent shear stress on meniscal tissue [23]. Indeed, the gel sheath that coats the TAPS sutures swells with the chitosan solution until it fills the entire circumference of the puncture hole in the meniscus. In this way, adhesion is achieved at the interface between the gel and the meniscal tissue [24]. The stress at the meniscus–suture interface is lower with TAPS sutures, and it induces less tissue damage than with pristine sutures (Figure 5) [23, 24]. Hopefully, these benefits can be correlated with better meniscal healing.

Furthermore, it is also interesting to note that the standard deviation was significantly smaller with the TAPS sutures compared to standard sutures, meaning that the behaviour of the TAPS sutures under cyclic tensile testing was consistent between specimens.

Further studies are required before these sutures can be used in humans, to determine their degradation pattern as well as their complex biomechanical properties. Also, an ex vivo study demonstrating that these augmented sutures could also decrease inflammation by reducing the surrounding acidity has recently been published [26].

Limitations of this study include its cadaveric design as well as the differences in size and stiffness between human and bovine menisci. Bovine menisci are bigger and stiffer than human ones, which could potentially lead to different results. Furthermore, the one‐stitch configuration used in the study does not correspond to what is usually done in human knees, which could also have an impact. However, as this study is a proof‐of‐concept, a simple suture was used to confirm the potential of TAPS suture and limit the risk of technical bias. In the future, more suture and meniscus tear patterns will be tested. There is also the possibility of a measurement bias when measuring the size of the suture loop. Moreover, cyclic tensile loading does not exactly mimic the forces experienced by a meniscus during human load bearing; an in vivo study will be needed in order to overcome this limitation.

## CONCLUSION

This biomechanical study demonstrates the effectiveness of hydrogel adhesive‐coated sutures (TAPS), favouring a better stress dispersion dissipation on the meniscal tissue, thus reducing the indentation caused by conventional sutures. These findings suggest that the use of these adhesives could improve outcomes in meniscal repair by reducing tissue damage and promoting healing.

## AUTHOR CONTRIBUTIONS

*Testing, analysis and interpretation of data and writing of the manuscript*: Stéphanie Lamer. *Testing, analysis and interpretation of data, creating figures and critical revision of the manuscript*: David Mazy. *Contributions to the research design, study supervision and data interpretation as well as critical revision*: Marie‐Lyne Nault.

## CONFLICT OF INTEREST STATEMENT

Marie‐Lyne Nault: The institution (Hopital Sacré‐Coeur de Montréal) has received departmental funding for research and educational purposes from: Arthrex, Conmed, Depuy, Linvatec, Smith & Nephew, Stryker, Synthes, Tornier, Wright, Zimmer Biomet. Departmental funding was also provided to CHU Sainte‐Justine from Orthopaediatrics and Smith and Nephew. This project did not receive any funding from these entities, and they were not involved in any aspect of the submitted work. The remaining authors declare no conflict of interest.

## ETHICS STATEMENT

The authors have nothing to report.

## Data Availability

Data available from the corresponding author upon reasonable request.
